# Enhancing the Performance of LLO Through Vanadium Doping and Abundant Exposed (010) Planes in Secondary Particles

**DOI:** 10.3390/nano15131017

**Published:** 2025-07-01

**Authors:** Shenghua Yuan, Chengwen Ren, Ziwei Liu, Yu Chen, Wenhui Wang

**Affiliations:** 1 College of Chemistry and Materials Science, Langfang Normal University, 100 Aimin West Road, Langfang 065000, China; yuchen@iccas.ac.cn; 2 Key Laboratory of Display Materials and Photoelectric Devices (MOE), School of Materials Science and Engineering, Tianjin University of Technology, 391 Binshui Road, Tianjin 300384, China; 15822639626@163.com; 3 China Petroleum Pipeline Engineering Corporation, 146 Heping Road, Langfang 065000, China; liu_ziwei@cnpc.com.cn

**Keywords:** lithium-ion battery, lithium-rich, V doping, (010) plane

## Abstract

Lithium-rich layered oxide (LLO) has received extensive attention from researchers due to its high initial discharge capacity (≥250 mAh g^−1^). However, defects such as its high initial irreversible capacity, voltage decay, and poor rate performance have severely limited its commercialization. These issues arise because the Li_2_MnO_3_ component in LLO is activated during the initial cycle, leading to the participation of lattice oxygen anions (O^2−^) in redox reactions. This results in irreversible oxygen loss (O_2_) and subsequent structural phase transitions. To address these challenges, this study focuses on Li_1_._2_Ni_0_._13_Co_0_._13_Mn_0_._54_O_2_ as the host material, utilizing abundant exposed (010) plane secondary particles and employing a vanadium (V) doping strategy to enhance electrochemical performance. The V forms strong V-O bonds with the lattice oxygen, effectively suppressing irreversible oxygen loss and improving structural stability. The results demonstrate that the LLO achieves the best electrochemical performance as the doping amount is 1 mol%, and the capacity retention improves from 74.5% (undoped) to 86% (V-doped) after 140 cycles at 0.5 C.

## 1. Introduction

Since energy density is a key limitation of lithium-ion batteries, enhancing the performance of cathode materials is crucial for battery development. However, commercial cathode materials such as LiCoO_2_ and LiFePO_4_ still fall short of the required energy density for future applications [1,2,3].

Lithium-rich layered oxide (1 − x)Li_2_MnO_3_·xLiMO_2_ (M = Ni, Co, Mn) has attracted extensive attention from researchers due to its excellent initial discharge capacity (>250 mAh g^−1^) and is regarded as a promising next-generation cathode material [4,5,6]. LLO operates within a voltage window of 2.0–4.8 V. During the initial charging process, Ni^2+^ and Co^3+^ in the LiMO_2_ component participate in the oxidation reaction and provide capacity as the voltage is within the range of 2–4.4 V (Ni^2+^/Ni^4+^, Co^3+^/Co^4+^) [7]. When the voltage exceeds 4.4 V, the Li_2_MnO_3_ component activates, with lattice oxygen anions (O^2−^) participating in redox reactions [8]. In this process, O^2−^ is oxidized to O^−^, contributing extra capacity. However, some O^2−^ is further oxidation to O_2_, leading to a structural transition from a layered to a spinel phase. This oxygen evolution leads to decreased initial Coulombic efficiency, voltage decay, and reduced cycling stability [9].

To solve the problem, researchers have tried many modification methods to improve the performance of LLO. Currently, the popular modification methods mainly include doping, coating, pretreatment, structural optimization design, etc. [10,11,12,13,14,15,16]. Among these, ion doping is a traditional and effective modification method. This approach works by introducing other ions to inhibit the irreversible lattice oxygen loss, stabilize the crystal structure, and suppress voltage decay. Notably, doping at different lattice sites can yield distinct effects. For example, Liu et al. improved the rate performance of LLO through Na doping at the Li site [10]. Density functional theory (DFT) calculations reveal that the introduction of Na^+^ expands the interlayer distance of Li_1.2_Mn_0.6_Ni_0.2_O_2_, reduces the band width, decreases the cation mixing phenomenon, and improves the Li^+^ diffusion rate and electronic conductivity. Yalçin, A et al. reported a one-pot approach for preparing Sn-doped Li_1.2_Mn_0.52-x_Sn_x_Ni_0.2_Co_0.08_O_2_ cathode materials through a supercritical-CO_2_-assisted method, which exhibited an excellent structural stability and rate performance [11].

Building on these doping strategies, high-valent V^5+^ doping presents unique advantages. V^5+^ can form V-O bonds with lattice oxygen anions, effectively inhibiting the irreversible lattice oxygen loss during the initial cycle process [17]. Moreover, the similar ionic radii of V^5+^ (0.54 Å) and Mn^4+^ (0.53 Å) indicate that V doping may expand interlayer spacing, facilitating accelerated Li^+^ diffusion and an improved rate performance. In a previous work, Lu et al. synthesized V-doped LLO through the low-temperature solid-phase thermal decomposition method and achieved a good electrochemical performance [17]. However, although V doping has been confirmed to enhance the electrochemical performance of LLO, there are relatively few related reports on direct V doping through co-precipitation methods. This limitation stems from the hydrolysis tendency of soluble vanadium salts like VCl_3_ in aqueous solutions, requiring acidic additives that complicate conventional carbonate co-precipitation processes [18,19,20]. Crucially, hydrochloric acid additives risk corroding carbonate precursors, compromising material quality. Thus, developing a reliable V doping method for co-precipitation is still an outstanding challenge.

To address this limitation, some researchers proposed to prepare LLO by controlling the hydroxide co-precipitation synthesis conditions and using high-temperature solid-phase sintering [21]. The LLO synthesized by this method has abundant exposed (010) planes at the interface, which can provide channels for Li^+^ diffusion and effectively improve the rate performance [21,22,23]. Importantly, the high-pH environment of hydroxide co-precipitation minimizes the impact of HCl, effectively overcoming the hydrolysis issue of vanadium salts.

Based on these insights, this study designs a V doping strategy using hydroxide co-precipitation combined with high-temperature solid-phase sintering to synthesize lithium-rich oxide with abundant exposed (010) planes. The effect of V doping on the structural evolution of LLO during the initial cycle is investigated. In addition, the electrochemical performance of V-doped LLO with abundant exposed (010) planes is systematically discussed.

## 2. Materials and Methods

### 2.1. Material Preparation

The pristine LLO and V-doped LLO are synthesized via a combination of hydroxide co-precipitation and solid-state sintering.

**Scheme 1:**
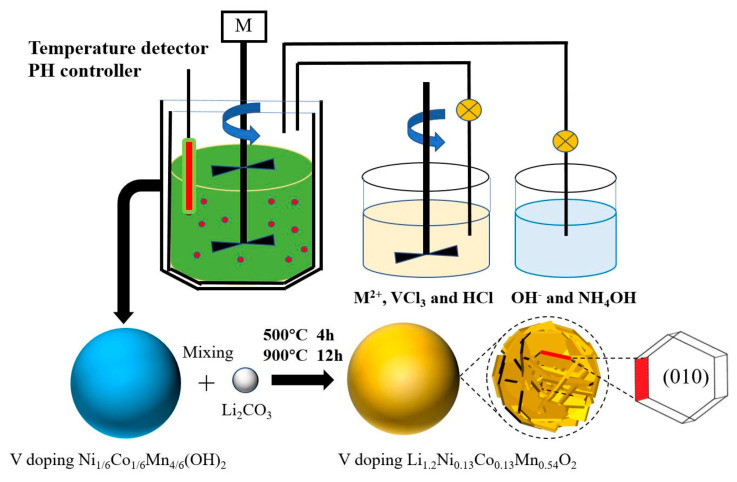
Preparation process of V-doped Li_1.2_Ni_0.13_Co_0.13_Mn_0.54_O_2_.

The pristine Ni_1_/_6_Co_1_/_6_Mn_4_/_6_(OH)_2_ precursors and V-doped Ni_1_/_6_Co_1_/_6_Mn_4_/_6_(OH)_2_ precursors were prepared by hydroxide co-precipitation. Weigh 263 g of NiSO_4_·6H_2_O (Aladdin, >99%), 281 g of CoSO_4_·7H_2_O (Aladdin, >99%), and 676 g of MnSO_4_·H_2_O (Aladdin, >99%), then dissolve them in deionized water to prepare a 3 L aqueous solution. This solution is fed into a continuously stirred tank reactor (CSTR) at a rate of 100 mL h^−1^. A mixture of sodium hydroxide (Aladdin, >99%, 10 mol L^−1^) and ammonium hydroxide (Aladdin, GR, 25%, 1.5 mol L^−1^) served as the precipitant and complexing agent. For V-doped samples, weigh 9.438 g and 18.876 g of VCl_3_ (Macklin, >99%), corresponding to 1 mol% and 2 mol% V-doped samples, respectively, and dissolve them in the transition metal solution, with a small amount (40 mL/80 mL) of dilute hydrochloric acid (Laboratory preparation, 20% concentration) added to suppress hydrolysis. The reaction conditions are maintained at pH of 10.75, 50 °C, and a stirring speed of 600 rpm [24]. The preparation process is shown in Scheme 1. The synthesized precursor was thoroughly washed with deionized water and subsequently dried at 60 °C. After drying, the precursor was sieved before being transferred to a muffle furnace for solid-state reaction. For cathode material preparation, either the pristine or V-doped precursor was mixed with stoichiometric amounts of Li_2_CO_3_ (Aladdin, >99%). The molar ratio of Li_2_CO_3_ to the precursor is 1.55:1. This is followed by a two-step sintering process in the muffle furnace: first at 500 °C for 4 h, then at 900 °C for 12 h to obtain the final materials. To facilitate description, the Li_1.2_Ni_0.13_Co_0.13_Mn_0.54_O_2_ materials with V doping amounts of 1 mol% and 2 mol% were labeled as LLO-V1% and LLO-V2%, respectively.

In this paper, the carbonate precursors were used in comparative experiments, and the preparation process has been reported in our previous study in the literature [25].

### 2.2. Material Characterization

The crystal structure of the samples was analyzed using an X-ray diffractometer (Rigaku D/Max-2500, Rigaku Corporation, Tokyo, Japan, Cu Kα radiation, 40 kV, 40 mA). Field-emission scanning electron microscopy (FE-SEM, JMS-6700F, JEOL, Tokyo, Japan) and transmission electron microscopy (TEM, TECNAI F-30, 300 kV, FEI, Agawam, MA, USA) were employed to characterize the morphology, crystallographic parameters, and microstructure of the samples. The elemental distribution mapping was characterized by energy dispersive spectrometers (EDSs) equipped with SEM. The valence states of Ni, Co, and Mn were characterized by X-ray photoelectron spectroscopy (XPS, Thermo Fisher K-Alpha, FEI, Agawam, MA, USA) with monochromatic Al Kα X-ray source. The thermal analysis of the precursor powder was carried out using a thermal analyzer (SDT650, Ta Instruments, New Castle, DE, USA) at a heating rate of 10 °C min^−1^ in air up to 1000 °C.

### 2.3. Electrochemical Measurements

CR2032 coin-type cells were assembled in an argon-filled glove box with water and oxygen content below 0.1 ppm to evaluate the electrochemical performance of the synthesized samples. Each half-cell consisted of a cathode, lithium plate anode, Celgard 2500 separator, and commercially obtained electrolyte (model: LB-372; purchase website: dodochem.net).

The cathode was prepared by uniformly mixing the active material (80 wt%), PVDF binder (10 wt%, Macklin), and Super P carbon black (10 wt%, Aladdin) at room temperature (25 °C) for 3 h. The resulting slurry was coated onto aluminum foil using a scraper, followed by drying it in a vacuum oven at 100 °C for 12 h. The dried foil was then rolled and punched into 12 mm diameter disks, with an active material loading mass of approximately 2.2–2.8 mg cm^−2^.

Electrochemical testing was performed within a voltage range of 2.0–4.8 V at a current density of 1 C (1C = 200 mA g^−1^, 25 °C). Lithium-ion diffusion kinetics were analyzed by electrochemical impedance spectroscopy (EIS) using an electrochemical workstation (PARSTAT 4000A, Berwyn, PA, USA) over a frequency range of 100 kHz to 0.01 Hz.

## 3. Results

**Figure 1:**
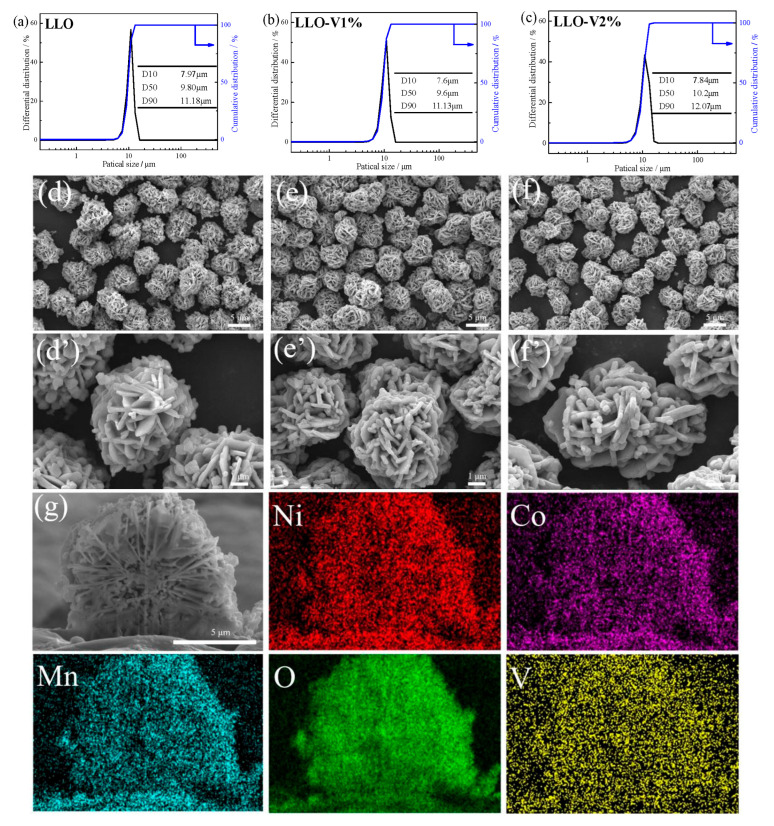
Particle size distribution of (**a**) LLO, (**b**) LLO-V1%, and (**c**) LLO-V2%. SEM images of (**d**,**d’**) LLO, (**e**,**e’**) LLO-V1%, and (**f**,**f’**) LLO-V2% samples. (**g**) Cross-section SEM image and mapping of LLO-V1% sample.

The particle size distributions of the samples before and after V doping are displayed in Figure 1a–c. As shown in the figures, all three samples exhibit relatively uniform particle size distributions. The median particle sizes (D_50_) of the LLO, LLO-V1%, and LLO-V2% samples are 9.8 μm, 9.6 μm, and 10.2 μm, respectively, suggesting that V doping has no significant impact on secondary particle size. The particle sizes of the secondary particles are all maintained within a reasonable range, so the interference of particle size with performance can be excluded. The SEM images of the LLO, LLO-V1%, and LLO-V2% samples are shown in Figure 1d–f. The secondary particles of LLO prepared by the hydroxide co-precipitation method are self-assembled from primary nanoplates, showing a significantly different morphology compared to those prepared by carbonate co-precipitation [25]. To investigate V doping, we performed a focused ion beam (FIB) treatment on the LLO-V1% sample and carried out elemental mapping on the cross-section of secondary particles (Figure 1g). The results demonstrate a relatively uniform distribution of all elements. Furthermore, Table 1 shows that the elemental content ratios match the designed composition, confirming the successful preparation of V-doped LLO materials.

**Figure 2:**
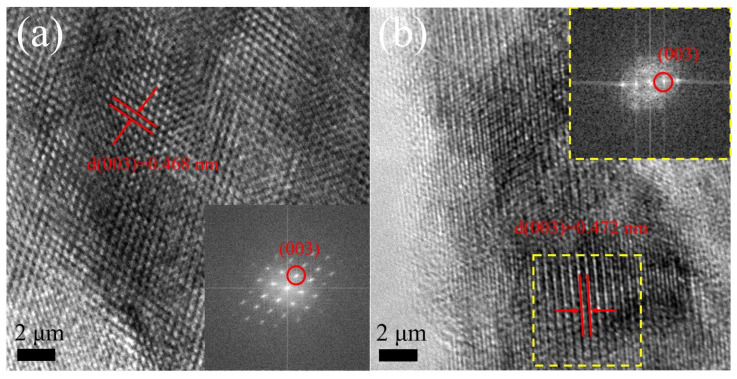
TEM images the corresponding Fourier transform of (**a**) LLO and (**b**) LLO-V1% samples.

Transmission electron microscopy (TEM) serves as a powerful tool for crystal structure characterization at the atomic scale. This technique provides a direct visualization of lattice arrangements through high-resolution imaging and Fourier transform analysis. As shown in the TEM results (Figure 2), the measured lattice fringe widths of 0.468 nm for the LLO and 0.472 nm for the LLO-V1% samples correspond to the (003) plane, as confirmed by their respective Fourier transforms. These findings demonstrate that the material maintains its well-ordered layered structure after vanadium doping [25,26,27]. While TEM offers exceptional structural insights, certain experimental limitations should be noted. The primary nanoplates’ orientation makes direct observation of the (010) plane particularly challenging under standard TEM conditions. However, we have previously addressed this characterization gap in our earlier work [24,28], in which we successfully analyzed the exposed (010) plane of secondary particles. Given these established findings and the technical constraints mentioned above, we have chosen not to repeat the (010) plane characterization in this paper. This decision allows us to focus our analytical efforts on the novel aspects of V-doped LLO materials while building upon our previously validated results.

**Figure 3:**
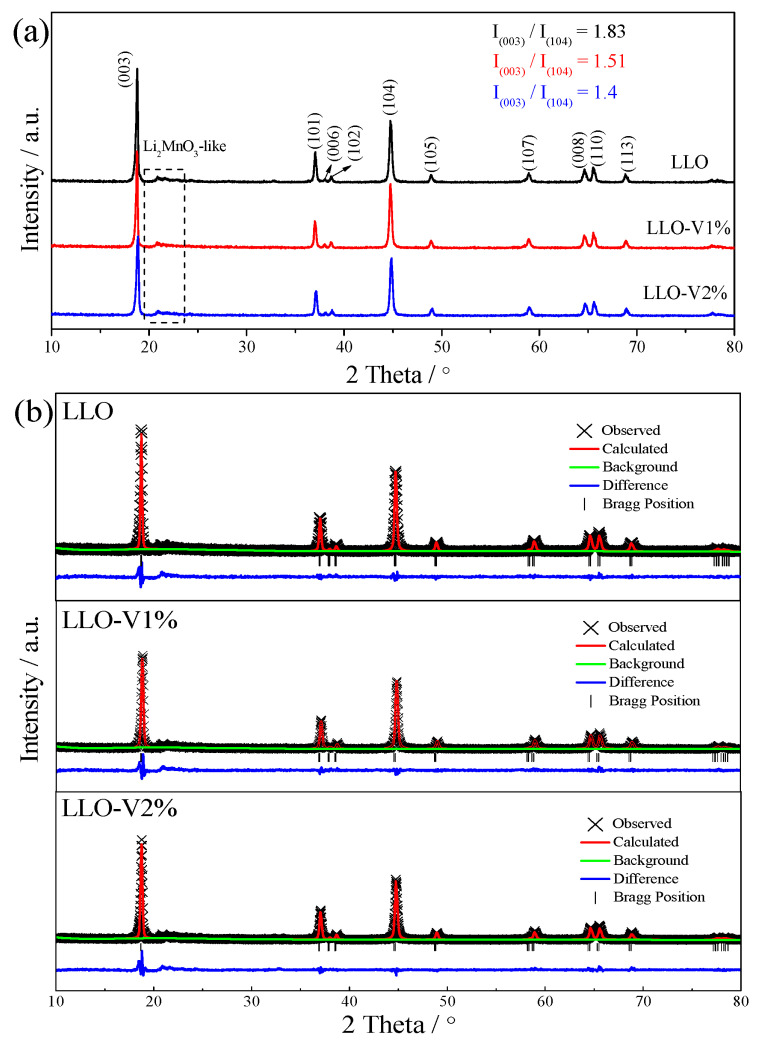
XRD (**a**) and Rietveld refinement results (**b**) of LLO, LLO-V1%, and LLO-V2%.

In this study, XRD patterns are used to characterize the structural information of LLO before and after V doping. In Figure 3a, the small peak around 20° represents the Li_2_MnO_3_ component of the lithium-rich material, which is consistent with that in the previous literature [29,30,31]. The (006) and (102) peaks of the LLO-V1% and LLO-V2% samples still exist, and the splitting degrees of the (108) and (110) peaks are relatively good, which indicates that the LLO still maintains a good layered structure after V doping. Furthermore, according to previous literature reports, I_(003)_/I_(104)_ can represent the orderliness of layered structures [24,25]. Interestingly, the data demonstrate a gradual decrease in this ratio with increasing vanadium content, indicating progressive structural disordering. This phenomenon likely stems from the ionic size mismatch between V^5+^ (0.54 Å) and Mn^4+^ (0.53 Å), in which the slightly larger vanadium ions introduce local lattice strain that disrupts the ideal layered arrangement.

The Rietveld refinement of the XRD results was conducted using the GSAS software to obtain further structural information. The refinement results are presented in Figure 3b and Table 2. As shown in Table 2, the discrepancy factors (R_p_ and R_wp_) fall within a reasonable range, confirming the reliability of the refined data. The table also reveals that the unit cell volume expands with the increasing V doping content, which can be attributed to the slightly larger ionic radius of V^5+^ compared to Mn^4+^. Additionally, the c/a ratio reflects the deviation of the crystal lattice from cubic symmetry [28]. As the V doping amount rises, the c/a ratio gradually decreases, suggesting a reduction in structural orderliness. This observation aligns with the trend observed in Figure 3a. Consequently, excessive V doping may destabilize the lattice structure, potentially leading to its collapse.

**Figure 4:**
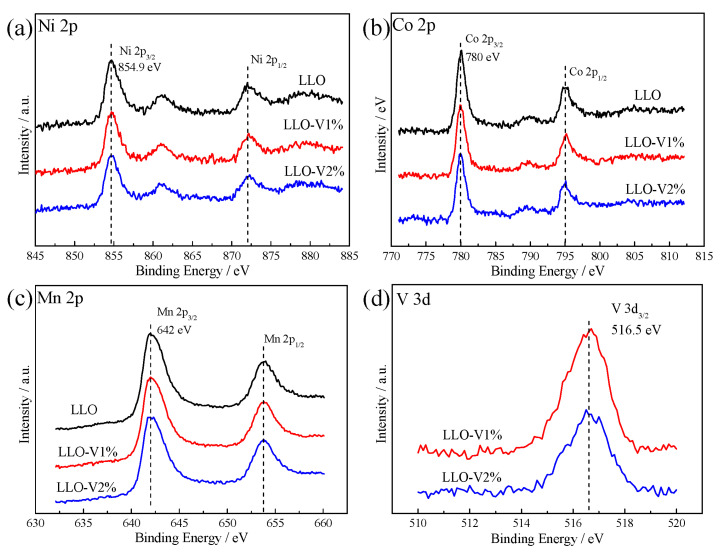
XPS spectra of (**a**) Ni 2p, (**b**) Co 2p, and (**c**) Mn 2p of LLO, LLO-V1%, and LLO-V2% samples. XPS spectra of (**d**) V 3d of LLO-V1% and LLO-V2% samples.

In this paper, the valence state information of each element in LLO is characterized by XPS. In Figure 4a–c, Ni 2p_3/2_, Co 2p_3/2_, and Mn 2p_3/2_ are located at 854.9 eV, 780 eV, and 642 eV, corresponding to Ni^2+^, Co^3+^, and Mn^4+^, respectively [32,33]. The existence of the V 3d peak in Figure 4d further proves that the V element has been successfully doped. Furthermore, V 3d_3/2_ is located at 516.5 eV, which indicates that the valence state of the V element is +5 [17]. Combined with the results in Figure 1d and Table 1, it can be confirmed that V-doped LLO was successfully synthesized.

**Figure 5:**
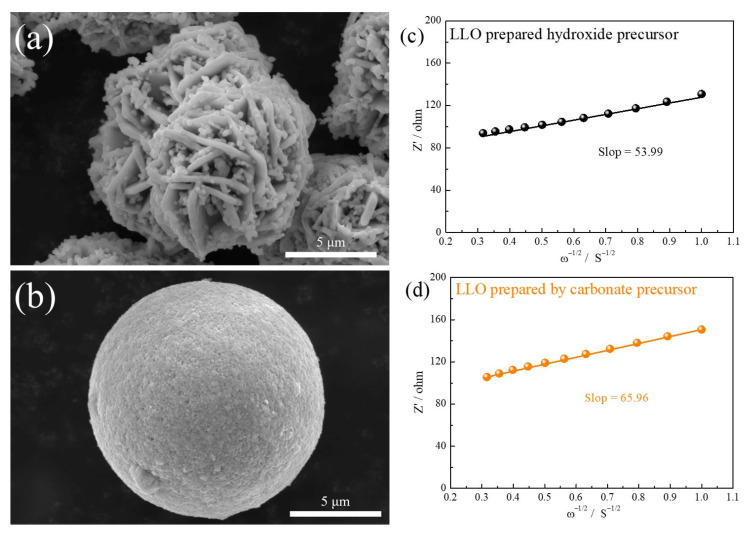
SEM images of (**a**) LLO prepared by hydroxide precursor and (**b**) LLO prepared by carbonate precursor. The relationship plots between Z’ and ω^−1/2^ derived from the low-frequency region of the EIS in the (**c**) LLO prepared by hydroxide precursor and (**d**) LLO prepared by carbonate precursor.

In addition, we employed LLO secondary particles with abundant exposed (010) planes as the host material for modification research. The performance advantages of these secondary particles were characterized and analyzed (Figure 5). Unlike LLO prepared from carbonate precursors, some researchers have successfully synthesized secondary particles with abundant exposed (010) planes by controlling hydroxide co-precipitation conditions. Previous studies have demonstrated that (010) planes provide efficient channels for Li^+^ diffusion [21,22,23]. Consequently, LLO secondary particles with abundant exposed (010) planes at the interface exhibit a superior rate performance. As shown in Figure 5a,b, significant morphological differences exist between lithium-rich materials synthesized from carbonate precursors and hydroxide precursors. The LLO prepared from the carbonate precursors consist of secondary particles formed by the aggregation of primary particles, and the relatively long transport paths between primary particles may hinder Li^+^ transport. In contrast, the LLO with abundant exposed (010) planes features secondary particles self-assembled from primary nanoplates. This unique structure enables rapid Li^+^ transport through the exposed (010) planes at the secondary particle interfaces. The ion diffusion coefficients of two LLO materials were tested. The diffusion coefficient of the lithium ion (*D*_Li_^+^) is calculated by the following formula:

(1) $$D_{{Li}^{+}}=\frac{R^{2}T^{2}}{2A^{2}n^{4}F^{4}C^{2}\sigma^{2}}$$

In the governing equation, *R* represents the ideal gas constant, *T* denotes the temperature in Kelvin, *A* stands for the electrode area, *n* indicates the number of electrons transferred during the reaction, *F* corresponds to the Faraday constant, and *C* signifies the lithium-ion bulk concentration [24,25]. These parameters collectively determine the ion transport characteristics of the system. To determine these parameters, the bulk concentration (*C*) is calculated through the ratio of the material density to the relative molecular weight of the active substance. This value serves as a critical input for subsequent diffusion analysis. Regarding the Warburg factor (*σ*), its determination involves a linear regression analysis between the real impedance component (Z’) and the inverse square root of frequency (ω^−1^/^2^), as illustrated in Figure 5c–d. The physical interpretation of these measurements reveals important trends. The inverse relationship between the Warburg factor and ion diffusion coefficient indicates that lower σ values correspond to enhanced ionic mobility. A comparative analysis demonstrates that the LLO synthesized via hydroxide precursors exhibits superior diffusion characteristics to its carbonate-derived counterpart, as evidenced by systematically lower *σ* values.

**Figure 6:**
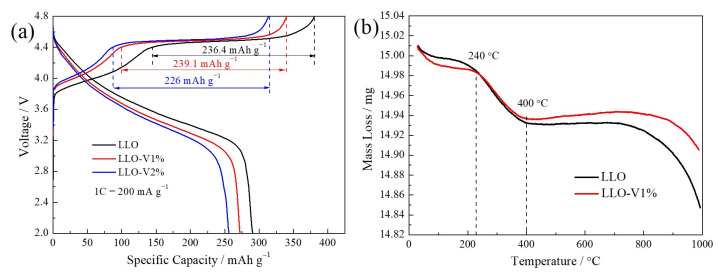
(**a**) Initial charge and discharge curves of LLO, LLO-V1%, and LLO-V2% samples. (**b**) TG curves of LLO and LLO-V1% samples.

According to previous literature reports, lattice oxygen anions in LLO participate in the oxidation reaction when the initial charging voltage exceeds 4.4 V. While this contributes significantly to its capacity, it also leads to irreversible lattice oxygen loss and structural phase transitions [34,35,36]. In this paper, the initial charge–discharge curves and thermogravimetric (TG) curves of LLO are used to evaluate the effect of V doping in suppressing irreversible lattice oxygen loss. As shown in Figure 6a, the discharge capacity of LLO gradually decreases with increasing V doping content. This phenomenon can be attributed to two main factors: 1. A reduction in the transition metal ion content: V doping decreases the proportion of redox-active transition metals (Ni, Co, and Mn), resulting in a lower capacity. 2. The stabilization of lattice oxygen: V forms strong V–O bonds with lattice oxygen anions, inhibiting their participation in the oxidation reaction during the initial cycle. Notably, the charging capacities in the 4.4–4.8 V range (236.4 mAh g^−1^, 239.1 mAh g^−1^, and 226 mAh g^−1^, respectively) indicate that a small amount of V doping does not significantly affect the lattice oxygen oxidation reaction. Furthermore, as shown in Table 3, the initial Coulombic efficiency of LLO improves with higher V doping amounts. In summary, although V doping slightly reduces the initial discharge capacity of LLO, it effectively suppresses irreversible lattice oxygen loss and enhances structural stability.

Furthermore, the suppression of irreversible lattice oxygen loss in V-doped LLO was investigated using a thermogravimetric (TG) analysis during the initial cycling process. The test samples consisted of mixed powders of the precursors Ni_1_/_6_Co_1_/_6_Mn_4_/_6_CO_3_ (pristine and 1 mol% V-doped) and Li_2_CO_3_. As shown in Figure 6b, the mixed powder gradually transitions to a molten state and loses 0.2% of its weight below 240 °C. A rapid weight loss occurs between 240 and 400 °C, attributed to the thermal decomposition of Li_2_CO_3_ and the Ni_1_/_6_Co_1_/_6_Mn_4_/_6_CO_3_ precursor (both pristine and V-doped), resulting in carbon removal. The plateau observed from 400 to 800 °C corresponds to the formation of LLO, while the weight loss between 800 and 1000 °C reflects oxygen loss during the sintering process [36]. Notably, the LLO-V1% sample exhibits better thermal stability than the undoped LLO, further confirming that V doping enhances lattice stability. This result aligns with the findings presented in Figure 6a.

**Figure 7:**
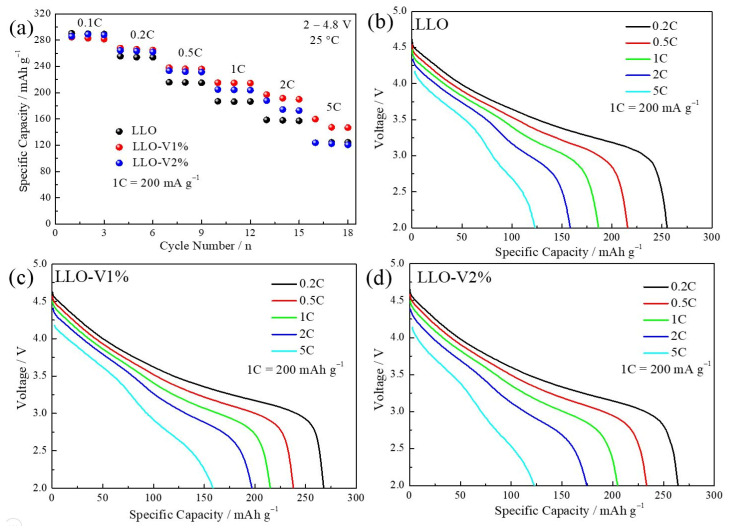
(**a**) Rate performance of LLO, LLO-V1%, and LLO-V2% samples. Discharge curves at different rates of (**b**) LLO sample, (**c**) LLO-V1% sample, and (**d**) LLO-V1% sample.

Figure 7 shows the rate performance of the LLO, LLO-V1%, and LLO-V2% samples. The results demonstrate that optimal rate performance is achieved with 1 mol% V doping in LLO. This is mainly attributed to two reasons. Firstly, as evidenced in Figure 5, the abundant exposed (010) planes at the LLO interface provide efficient channels for Li^+^ diffusion, significantly contributing to improved rate performance. On the other hand, the ionic radius of V^5+^ is slightly larger than that of Mn^4+^, and V doping effectively expands the interlayer spacing, thereby facilitating faster ion transport. The rate performance decreases as the doping amount of V increases to 2 mol%, which may be attributed to the lattice structure collapse caused by excessive doping.

**Figure 8:**
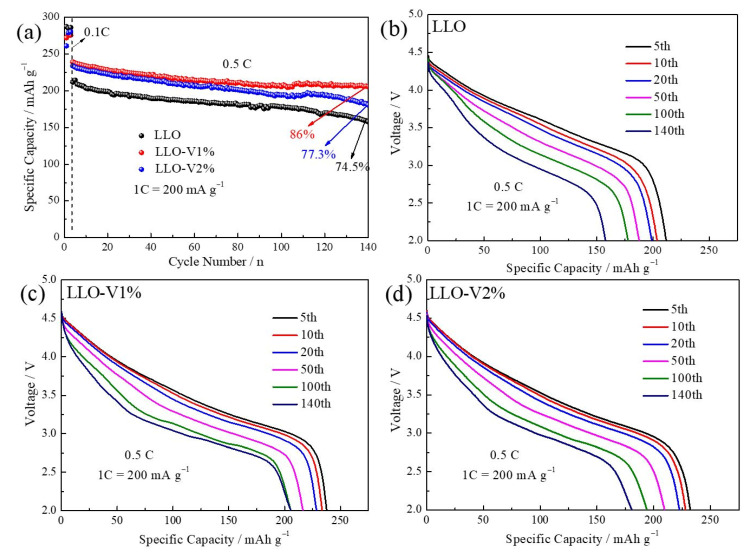
(**a**) Cycle performance of LLO, LLO-V1%, and LLO-V2% samples. Discharge curves for different cycles of (**b**) LLO sample, (**c**) LLO-V1% sample, and (**d**) LLO-V1% sample.

The cycling performance of LLO before and after V doping is shown in Figure 8. It can be seen from the figure that the LLO shows the best cycling stability with V doping at 1 mol%. The capacity retention from 74.5% increased to 86% after 140 cycles at 0.5 C. According to the results in Figure 6 and previous literature reports, this is mainly attributed to two factors. Firstly, V doping forms V-O with lattice oxygen in LLO, which can effectively suppress the irreversible loss of lattice oxygen during the initial cycling process [17,24,37]. On the other hand, according to previous literature reports, V doping can stabilize the lattice structure of LLO and enhance the cycling stability [17,38].

**Figure 9:**
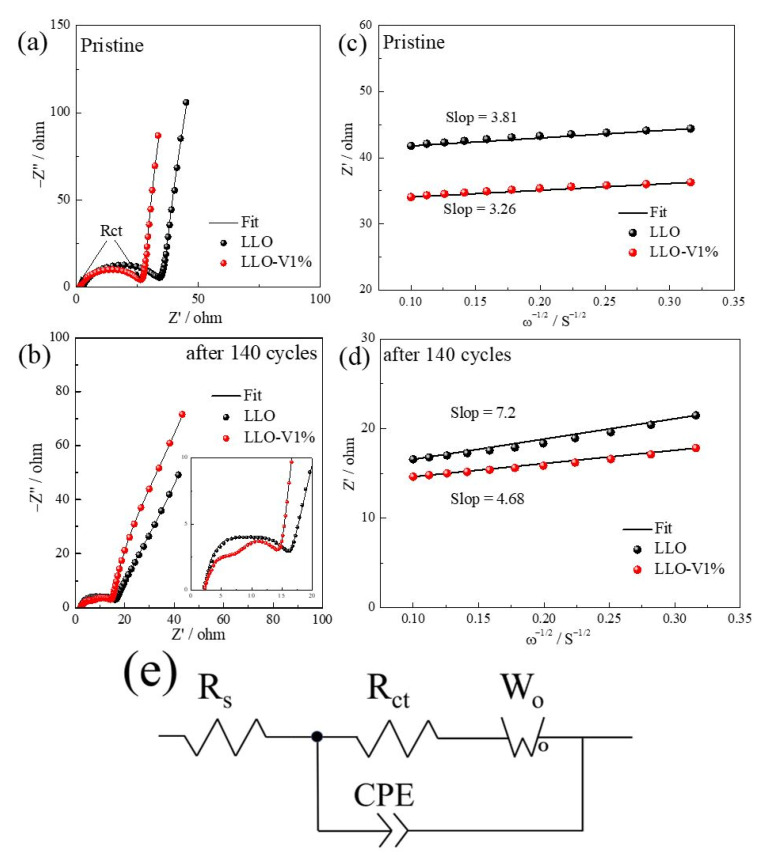
EIS of LLO and LLO-V1% samples in the (**a**) pristine condition and (**b**) after 140 cycles at 0.5 C. The relationship plots between Z′ and ω^−1/2^ of the (**c**) pristine condition and (**d**) after 1400 cycles. (**e**) Equivalent circuit diagram of ElS impedance spectrum.

Based on a previous analysis, the LLO demonstrates optimal electrochemical performance with 1 mol% V doping. To further investigate the effect of V doping on the electrochemical performance of LLO, we conducted EIS impedance spectroscopy measurements on both pristine LLO and LLO-V1% samples. The EIS data are analyzed using ZsimpWin software, with fitting results presented in Table 4. In Table 4, R_s_ represents the ohmic resistance of the sample, and R_ct_ corresponds to the charge transfer resistance, manifested as a semicircle in the high-frequency region of the Nyquist plot [39]. Figure 9a shows that the pristine LLO exhibits higher charge transfer resistance than the V-doped sample, attributable to both the larger ionic radius of V^5+^ relative to Mn^4+^ and the enhanced V doping Li^+^ diffusion. Figure 9b presents the impedance spectra after 140 cycles, showing that LLO-V1% maintains a lower charge transfer resistance than the undoped sample, which is attributed to the fact that V doping can stabilize the lattice structure of LLO. This is consistent with the results of Figure 6 and Figure 8. Based on Warburg impedance data, the diffusion coefficient of the lithium ion (*D*_Li_^+^) is further calculated by Equation (1). According to our calculations, the Li^+^ diffusion coefficients of the LLO and LLO-V1% samples in the initial state are 5.19 × 10^−11^ and 7.08 × 10^−11^, respectively. And the Li^+^ diffusion coefficients of the LLO and LLO-V1% samples after 140 cycles are 1.45 × 10^−11^ and 3.44 × 10^−11^, respectively. Therefore, the lithium-rich layered oxides obtained higher Li^+^ diffusion coefficients both before and after V doping.

## 4. Conclusions

In this study, V-doped lithium-rich layered oxides (LLOs) with abundant exposed (010) planes are synthesized by modifying the hydroxide co-precipitation process followed by high-temperature solid-phase sintering. The results demonstrate that V doping effectively suppresses the irreversible capacity loss during the initial cycling process and improves cycling stability. Furthermore, the modification is applied to secondary particles with abundant exposed (010) planes as the host material, leveraging their ability to accelerate Li^+^ diffusion and thereby enhance the rate performance of LLO. By combining these two modification strategies, the LLO achieves a superior electrochemical performance due to their synergistic effect.

## Figures and Tables

**Table 1:** Cross-section mapping characterization element data of 1% molar ratio V-doped Li_1.2_Ni_0.13_Co_0.13_Mn_0.54_O_2_.

Element	Apparent Concentration	K Ratio	Wt (%)	Atomic Percentage (%)
O	9.90	0.03330	29.56	60.10
Mn	4.70	0.04700	44.96	26.62
Co	1.16	0.01161	11.55	6.38
Ni	1.22	0.01219	11.77	6.52
V	0.17	0.00170	2.15	0.38

**Table 2:** Lattice parameters and analysis of X-ray Rietveld refinement results before and after V-doped Li_1.2_Ni_0.13_Co_0.13_Mn_0.54_O_2_.

Sample	a (Å)	c (Å)	c/a	V (Å^3^)	R_p_ (%)	R_wp_ (%)
LLO	2.8504	14.2359	4.9944	100.17	8.08	4.14
LLO-V1%	2.8547	14.2570	4.9942	100.62	8.69	5.30
LLO-V2%	2.8581	14.2721	4.9936	100.96	9.21	6.23

**Table 3:** The electrochemical performance of LLO, LLO-V1%, and LLO-v2% samples.

Sample	Initial Charging Capacity (mAh g^−1^)	Initial Discharge Capacity (mAh g^−1^)	Initial Coulomb Efficiency (%)	Capacity Retention (%)
LLO	380.5	290.8	76.4	74.5
LLO-V1%	339.8	272.2	80.1	86
LLO-V2%	313.7	256	81.6	77.3

**Table 4:** Impedance data of LLO sample and LLO-V1% sample before and after 140 cycles.

Sample	Rs/ohm	Rct/ohm	*D*_Li_^+^/cm^2^ s^−1^
LLO	2.7	34.8	5.19 × 10^−11^
LLO-V1%	1.5	26.4	7.08 × 10^−11^
LLO-140cycles	2.3	16.1	1.45 × 10^−11^
LLO-V1%-140cycles	2.2	14.1	3.44 × 10^−11^

## Data Availability

The original contributions presented in the study are included in the article; further inquiries can be directed to the corresponding authors.

## References

[1] Tarascon J.M., Armand M. (2001). Issues and challenges facing rechargeable lithium batteries. Nature.

[2] Dunn B., Kamath H., Tarascon J.M. (2011). Electrical Energy Storage for the Grid: A Battery of Choices. Science.

[3] Chen Z.L., Zhang Q., Liang Q.J. (2022). Carbon-Coatings Improve Performance of Li-Ion Battery. Nanomaterials.

[4] Nie L., Chen S.J., Liu W. (2023). Challenges and strategies of LLO for Li-ion batteries. Nano Res..

[5] Zhao S.Q., Yan K., Zhang J.Q., Sun B., Wang G.X. (2021). Reaction Mechanisms of Layered Lithium-Rich Cathode Materials for High-Energy Lithium-Ion Batteries. Angew. Chem.-Int. Ed..

[6] Lu H.Y., Hou R.L., Chu S.Y., Zhou H.S., Guo S.H. (2023). Progress on Modification Strategies of Layered Lithium-Rich Cathode Materials for High Energy Lithium-Ion Batteries. Acta Phys.-Chim. Sin..

[7] Thackeray M.M., Kang S.H., Johnson C.S., Vaughey J.T., Benedek R., Hackney S.A. (2007). Li_2_MnO_3_-stabilized LiMO_2_ (M = Mn, Ni, Co) electrodes for lithium-ion batteries. J. Mater. Chem..

[8] Carroll K.J., Qian D., Fell C., Calvin S., Veith G.M., Chi M.F., Baggetto L., Meng Y.S. (2013). Probing the electrode/electrolyte interface in the lithium excess layered oxide Li_1.2_Ni_0.2_Mn_0.6_O_2_. Phys. Chem. Chem. Phys..

[9] Gu M., Belharouak I., Zheng J.M., Wu H.M., Xiao J., Genc A., Amine K., Thevuthasan S., Baer D.R., Zhang J.G. (2013). Formation of the Spinel Phase in the Layered Composite Cathode Used in Li-Ion Batteries. Acs Nano.

[10] Liu S.N., Yan X., Li P.Y., Tian X.R., Li S.N., Teng F., Luo S.H. (2024). Structural and enhanced electrochemical performance of Co-free lithium-rich layered manganese-based Li_1.2_Mn_0.6_Ni_0.2_O_2_ cathodes via Na-doping at Li site for lithium-ion batteries. Mater. Today Sustain..

[11] Yalçin A., Demir M., Güler M.O., Gönen M., Akgün M. (2023). Synthesis of Sn-doped Li-rich NMC as a cathode material for Li-ion batteries. Electrochim. Acta.

[12] Makhonina E., Pechen L., Medvedeva A., Politov Y., Rumyantsev A., Koshtyal Y., Volkov V., Goloveshkin A., Eremenko I. (2022). Effects of Mg Doping at Different Positions in Li-Rich Mn-Based Cathode Material on Electrochemical Performance. Nanomaterials.

[13] Shevtsov A., Han H.X., Morozov A., Carozza J.C., Savina A.A., Shakhova I., Khasanova N.R., Antipov E.V., Dikarev E.V., Abakumov A.M. (2020). Protective Spinel Coating for Li_1.17_Ni_0.17_Mn_0.50_Co_0.17_O_2_ Cathode for Li-Ion Batteries through Single-Source Precursor Approach. Nanomaterials.

[14] Ge H., Bai J.S., Wang C.Y., Xie L.H., Li W.F., Sun Z.J., Cao X.M. (2025). Advanced surface engineering of lithium-rich manganese-based cathodes towards next-generation lithium-ion batteries. J. Energy Chem..

[15] Qiu B., Zhang M.H., Wu L.J., Wang J., Xia Y.G., Qian D.N., Liu H.D., Hy S., Chen Y., An K. (2016). Gas-solid interfacial modification of oxygen activity in layered oxide cathodes for lithium-ion batteries. Nat. Commun..

[16] Yuan M.M., Wang L.D., Zhang J., Ran M.J., Wang K., Hu Z.Y., Van Tendeloo G., Li Y., Su B.L. (2024). Cut-off voltage influencing the voltage decay of single crystal lithium-rich manganese-based cathode materials in lithium-ion batteries. J. Colloid Interface Sci..

[17] Zhou M.M., Zhao J.J., Wang X.D., Shen J., Yang J.L., Tang W.H., Deng Y.R., Zhao S.X., Liu R.P. (2022). Enhanced stability of vanadium-doped Li_1.2_Ni_0.16_Co_0.08_Mn_0.56_O_2_ cathode materials for superior Li-ion batteries. RSC Adv..

[18] Hu W.H., Zhang Y.X., Zan L., Cong H.J. (2020). Mitigation of voltage decay in Li-rich layered oxides as cathode materials for lithium-ion batteries. Nano Res..

[19] Wu T.H., Liu X., Zhang X., Lu Y., Wang B.Y., Deng Q.S., Yang Y.B., Wang E.R., Lyu Z.T., Li Y.Q. (2021). Full Concentration Gradient-Tailored Li-Rich Layered Oxides for High-Energy Lithium-Ion Batteries. Adv. Mater..

[20] Wang Y.Z., Liu S.Q., Guo X.W., Wang B.Y., Zhang Q.H., Li Y.Q., Wang Y.L., Wang G.Q., Gu L., Yu H.J. (2025). Elements gradient doping in Mn-based Li-rich layered oxides for long-life lithium-ion batteries. J. Mater. Sci. Technol..

[21] Chen L., Su Y.F., Chen S., Li N., Bao L.Y., Li W.K., Wang Z., Wang M., Wu F. (2014). Hierarchical Li_1.2_Ni_0.2_Mn_0.6_O_2_ nanoplates with exposed {010} planes as high-performance cathode material for lithium-ion batteries. Adv. Mater..

[22] Wei G.Z., Lu X., Ke F.S., Huang L., Li J.T., Wang Z.X., Zhou Z.Y., Sun S.G. (2010). Crystal habit-tuned nanoplate material of Li[Li_1/3-2x/3_Ni_x_Mn_2/3-x/3_]O_2_ for high-rate performance lithium-ion batteries. Adv. Mater..

[23] Fu F., Xu G.L., Wang Q., Deng Y.P., Li X., Li J.T., Huang L., Sun S.G. (2013). Synthesis of single crystalline hexagonal nanobricks of LiNi_1/3_Co_1/3_Mn_1/3_O_2_ with high percentage of exposed {010} active facets as high rate performance cathode material for lithium-ion battery. J. Mater. Chem. A.

[24] Yuan S.H., Zhang H.Z., Song D.W., Ma Y., Shi X.X., Li C.L., Zhang L.Q. (2022). Regulate the lattice oxygen activity and structural stability of LLO by integrated strategies. Chem. Eng. J..

[25] Yuan S.H., Guo J., Ma Y., Zhou Y., Zhang H.Z., Song D.W., Shi X.X., Zhang L.Q. (2021). Improving the Electrochemical Performance of a Lithium-Rich Layered Cathode with an In Situ Transformed Layered@Spinel@Spinel Heterostructure. ACS Appl. Energy Mater..

[26] Zhang H.Y., Dong J.Y., Chen G., Che H.Q., Yan K., Wang X., Liu J.Z., Liu D.W., Lu Y., Li N. (2025). Trifunctional surface engineering of Lithium-rich manganese-based oxides via Al^3+^/PO_4_^3–^ co-doping and oxygen vacancy regulation for High-performance lithium-ion batteries. Chem. Eng. J..

[27] Lin H.F., Cheng S.T., Chen D.Z., Wu N.Y., Jiang Z.X., Chang C.T. (2022). Stabilizing Li-Rich Layered Cathode Materials Using a LiCoMnO_4_ Spinel Nanolayer for Li-Ion Batteries. Nanomaterials.

[28] Yuan S.H., Wang W.H., Ren C.W., Liu Z.W., Chen Y., Zhang H.Z., Zhang L.Q. (2025). Investigation of Sn-Doped Lithium-Rich Layered Oxides Based on Self-Assembled Secondary Particles Composed of Exposed (010) Plane Nanoplates. Langmuir.

[29] Yang Y.L., Gao C., Luo T., Song J., Yang T.H., Wang H.C., Zhang K., Zuo Y.X., Xiao W.K., Jiang Z.W. (2023). Unlocking the Potential of Li-Rich Mn-Based Oxides for High-Rate Rechargeable Lithium-Ion Batteries. Adv. Mater..

[30] Peng Y., Wu L., Li C.F., Luo B.C., Feng X.Y., Hu Z.Y., Li Y., Su B.L. (2023). One-pot K plus and PO^a–^ co-doping enhances electrochemical performance of Li-rich Li_1.2_Ni_0.13_Co_0.13_Mn_0.54_O_2_ cathode for Li-ion battery. Electrochim. Acta.

[31] Duan J.D., Huang M.J., Yang M.X., Li S.M., Zhang G., Guo J.Q., Yue B., Tang C.Y., Liu H. (2023). Anion-Cation Dual-Ion Multisite Doping Stabilizes the Crystal Structure of Li-Rich Layered Oxides. ACS Appl. Mater. Interfaces.

[32] Zhang P.P., Zhai X.H., Huang H., Zhou J.F., Li X.B., He Y.P., Guo Z.C. (2020). Synergistic Na^+^ and F^−^ co-doping modification strategy to improve the electrochemical performance of Li-rich Li_1.20_Mn_0.54_Ni_0.13_Co_0.13_O_2_ cathode. Ceram. Int..

[33] He J.Y., Ma H.Y., Zhang H.Z., Song D.W., Shi X.X., Deng Q.B., Li C.L., Jiao L.F., Zhang L.Q. (2020). Promoting the Electrochemical Performance of Li-Rich Layered Li_1.2_(Ni_1/6_Co_1/6_Mn_4/6_)_0.8_O_2_ with the In Situ Transformed Allogenic Spinel Phase. ACS Sustain. Chem. Eng..

[34] Croy J.R., Gallagher K.G., Balasubramanian M., Chen Z.H., Ren Y., Kim D., Kang S.H., Dees D.W., Thackeray M.M. (2013). Examining Hysteresis in Composite xLi_2_MnO_3_·(1-x)LiMO_2_ Cathode Structures. J. Phys. Chem. C.

[35] Dogan F., Croy J.R., Balasubramanian M., Slater M.D., Iddir H., Johnson C.S., Vaughey J.T., Key B. (2015). Solid State NMR Studies of Li_2_MnO_3_ and Li-Rich Cathode Materials: Proton Insertion, Local Structure, and Voltage Fade. J. Electrochem. Soc..

[36] Mohanty D., Li J.L., Abraham D.P., Huq A., Payzant E.A., Wood D.L., Daniel C. (2014). Unraveling the Voltage-Fade Mechanism in High-Energy-Density Lithium-Ion Batteries: Origin of the Tetrahedral Cations for Spinel Conversion. Chem. Mater..

[37] Kang H.E., Park T.M., Song S.G., Yoon Y.S., Lee S.J. (2024). Optimization of LiNiCoMnO_2_ Cathode Material Synthesis Using Polyvinyl Alcohol Solution Method for Improved Lithium-Ion Batteries. Nanomaterials.

[38] Lu C., Yang S.Q., Wu H., Zhang Y., Yang X.J., Liang T.H. (2016). Enhanced electrochemical performance of Li-rich Li_1.2_Mn_0.52_Co_0.08_Ni_0.2_O_2_ cathode materials for Li-ion batteries by vanadium doping. Electrochim. Acta.

[39] Yuan S.H., Guo J., Ma Y., Zhang H.Z., Song D.W., Shi X.X., Zhang L.Q. (2021). Boosting the Electrochemical Performance of a Spinel Cathode with the In Situ Transformed Allogenic Li-Rich Layered Phase. Langmuir.

